# Association between antidiabetic agents use and leukocyte telomere shortening rates in patients with type 2 diabetes

**DOI:** 10.18632/aging.101781

**Published:** 2019-01-28

**Authors:** Juanhong Liu, Yuanlong Ge, Shu Wu, Delin Ma, Weijie Xu, Ye Zhang, Yan Yang

**Affiliations:** ^1^Department of Endocrinology, Tongji Hospital, Tongji Medical College, Huazhong University of Science and Technology, Wuhan, Hubei 430030, China; ^2^Key Laboratory of Gene Engineering of the Ministry of Education, School of Life Sciences, Sun Yat-sen University, Guangzhou, Guangdong 510006, China; ^*^Equal contribution

**Keywords:** aging, acarbose, antidiabetic agents, type 2 diabetes, telomere shortening rate

## Abstract

Telomere length and telomere shortening rate (TSR) are accepted indicators of aging in cross-sectional population studies. This study aimed to investigate the potential influence of common antidiabetic agents on telomere length and TSR in patients with type 2 diabetes mellitus (T2DM). Leukocyte telomere length was measured through terminal restriction fragment analysis, and TSR was calculated in 388 T2DM patients. Depending on whether or not they received antidiabetic medication, patients were first divided into a treatment group and a nontreatment group. Treated patients were further subdivided into an acarbose-free group (patients taking antidiabetic agents without acarbose) and an acarbose group (patients using acarbose for more than 3 months). Results showed that untreated patients had higher TSRs than patients on antidiabetic drugs. Interestingly, patients in the acarbose group had significantly higher TSRs than patients in the acarbose-free group. Compared to the nontreatment group, the acarbose group showed better glycemic control of HbA1c, but the TSR was also higher. Our results suggest that antidiabetic treatments without acarbose can slow aging. By contrast, acarbose may accelerate biological aging in patients with T2DM, independently of glycemic control.

## INTRODUCTION

Diabetes is a metabolic disease characterized by chronic hyperglycemia and altered glycolipid and protein metabolism. Approximately 110 million adults have been diagnosed with type 2 diabetes mellitus (T2DM) in China, and its prevalence has steadily increased from 1% in 1980 to 10.9% in 2013 [1]. Age and obesity, as well as genetic, epigenetic, and environmental factors all contribute to the development of T2DM [2]. Cellular aging is commonly linked to a chronic, subclinical inflammatory state [3]. As its prevalence increases in the elderly, T2DM is often considered an age-related disease.

Telomeres are particularly susceptible to age-related deterioration [4]. These structures are composed of a specialized sequence of double-stranded DNA repeats that preserve chromosome structure and functional stability [5]. Telomere length shortens over the course of human lifespan [6]. This limits cellular proliferation, damages cells, and ultimately shortens lifespan [7]. Telomere shortening is not only considered a marker of biological aging, but is often correlated with an increased risk of developing age-related diseases such as cancer, diabetes, and cardiovascular disease [8]. The telomere shortening rate (TSR) is commonly used to measure how telomere shortening progresses with age [9]. TSR is obtained simply from the slope of the linear regression line between telomere length and age [10, 11]. TSRs based on cross-sectional studies are consistent with those obtained from longitudinal studies, and increase with aging across populations [12, 13]. The reliability of TSR is related to robust methods of telomere length measurement [14]. Terminal restriction fragment (TRF) analysis and other techniques such as quantitative PCR (qPCR)-based methods are popular choices for telomere length detection in epidemiological studies [15–17]. While qPCR-based techniques are convenient, high throughput, and require little DNA [18], questions have been raised regarding its reliability, affected particularly by high interassay coefficients of variation. TRF analysis is considered the gold standard within telomere biology, as it provides data on absolute telomere length and heterogeneity, although this method requires a large quantity of DNA and is cumbersome, labor intensive, and costly [19]. However, compared with other methods, a lower sample quantity is needed to obtain accurate analysis [20–22]. Many studies have described the relationship between telomeres and diabetes, and suggested that telomere shortening contributes critically to its pathogenesis [23]. Peripheral blood leukocyte telomere shortening has been reported as an independent risk factor for the development of type 2 diabetes in American Indians and could be used as an important indicator for predicting its progression [24]. Testa *et al.* and others have reported that leukocyte telomere lengths are shortened in diabetic patients with complications [25, 26]. TSR in T2DM patients is mitigated by adequate glycemic control [27]. Acarbose, a fermentation byproduct of the soil microorganism Actinoplanes utahensis, inhibits alpha-glucosidase and polysaccharide digestion, reduces glucose absorption in the intestinal brush border, and is widely used in China as an anti-diabetic drug. Other agents commonly used to control glycemia in diabetes include metformin and sulfonylureas. Although many studies have examined the relationship between telomere dynamics and diabetes, very few have analyzed the effects of antidiabetic agents on absolute telomere length or TSR. Therefore, this study was designed to compare telomere length and TSR in T2DM patients with or without treatment, and whether these variables are influenced by different antidiabetic agents.

## RESULTS

### Untreated T2DM correlates with higher TSR

TRF analyses were performed in peripheral blood leukocytes to evaluate the association of treatment agents for T2DM with telomere length and TSR. The mean telomere length in our T2DM study population was 4268–8661 base pairs (bp), and TSR was 11–16 base pairs per year (bp/year). Leukocyte telomere length significantly shortened with age in all patients (Supplementary Figure 2).

**Figure 1:**
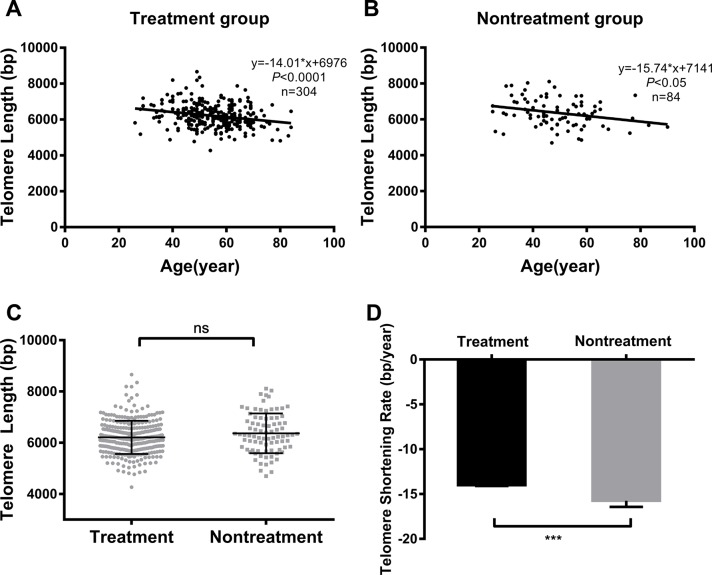
**Comparison of telomere length and TSR between treated vs untreated T2DM patients.** Linear regression analysis of telomere length and age in the treatment group (**A**) and nontreatment group (**B**); comparison of telomere length (**C**) and TSR (**D**) between the two groups. Solid lines in **A** and **B** indicate mean telomere lengths, calculated by regression analyses; y = -14.01*x+6976 in **A** (*r* = -0.24, *P* < 0.0001) and y = -15.74*x+7141 in **B** (*r* = -0.27, *P* < 0.05). y = telomere length in bp and x = age in years. (**D**) TSR was found to be -14.01 ± 3.28 bp/year in the treatment group (n = 304) and -15.74 ± 6.21 bp/year in the nontreatment group (n = 84). Telomere length is presented as the mean ± SD. TSRs are presented as the mean ± SEM. ns indicates *P* > 0.05, ***indicates *P* < 0.001

We divided diabetic patients into treatment and nontreatment groups, according to whether they were using antidiabetic agents for glycemic control or not. Linear regression analysis showed that telomere length shortened with age in both the treatment and nontreatment groups (Figure 1A and 1B). The correlation analysis showed that age was negatively related to telomere length in both groups (*r* = -0.24 and* r* = -0.27 for the treatment group and nontreatment group, respectively; all* P* < 0.05). Patients' characteristics are presented in Table 1. Patients who did not use antidiabetic agents exhibited higher levels of postprandial plasma glucose, glycated hemoglobin and fructosamine, and a greater incidence of acute complications than those using antidiabetic agents (all* P* < 0.05). The patients in the treatment group were older than those in the nontreatment group. However, telomere lengths in the two groups were almost the same (6210.08 ± 647.82 bp *vs.* 6360.04 ± 766.75 bp, treatment group *vs.* nontreatment group: *P* = 0.072; Figure 1C).

**Table 1:** Patient characteristics: Treatment vs nontreatment

Characteristic	Treatment group	Nontreatment group	*P*
Patients, n	304	84	-
Age, years	54.63±11.05	49.10±13.25	<0.0005
Males, n (%)	165 (54.3)	48 (57.1)	0.711
Fasting plasma glucose, mmol/L	9.71±3.98	10.47±5.82	0.163
Postprandial plasma glucose, mmol/L	16.81±6.32	18.70±5.78	0.026
HbA1c, (%)	8.76±2.30	10.57±2.86	<0.0005
Fructosamine, mmol/L	367.71±107.52	440.65±142.74	<0.0005
Chronic complications, n (%)	186 (61.2)	33 (39.3)	<0.0005
Acute complications, n (%)	13 (4.3)	11 (13.1)	0.008

Data are presented as mean ± SD, number, or percentage

*P* values were calculated using *Student’s t* test or χ2 test

Since TSR from cross-sectional studies is consistent with those obtained from longitudinal studies, we derived TSR from the slope of linear regression analysis. After adjustment for age, the TSR of the nontreatment group was significantly higher than that of the treatment group (-15.74 ± 6.21 bp/year *vs.* -14.01± 3.28 bp/year respectively, *P* < 0.001; Figure 1D).

### Acarbose treatment is associated with higher TSR

Glycemic control is a comprehensive process, in which antidiabetic agents are the most important element. In our preliminary study, we divided the patients into 4 groups: an insulin group, a metformin group, an acarbose group, and a sulfonylurea group. We found that the TSR varied among the four groups, but was the highest in patients receiving acarbose treatment (Supplementary Figure 3).

To further confirm the association between acarbose use and TSR, the treatment group was further subdivided into an acarbose-free group (patients taking antidiabetic agents without acarbose) and an acarbose group (patients using acarbose for more than 3 months). The baseline data of these two groups are shown in Table 2. Other than acarbose use, we observed no significant differences in the hypoglycemic regimens of the two groups. Fasting and postprandial serum glucose levels, HbA1C levels, fructosamine levels, and the incidence of acute complications did not significantly differ between the two groups. However, the prevalence of chronic complications was significantly greater in the acarbose-free group (χ^2^ = 7.153, *P* < 0.05).

**Table 2:** Patient characteristics: Acarbose-free vs acarbose

Characteristic	Acarbose-free group	Acarbose group	*P*
Patients, n	215	89	-
Age, years	54.8±10.4	54.2±12.5	0.439
Males, n (%)	113 (52.6)	52 (58.4)	0.377
Fasting plasma glucose, mmol/L	9.62±3.77	9.99±4.48	0.585
Postprandial plasma glucose, mmol/L	18.70±5.78	17.51±7.34	0.301
HbA1c, mmol/mol	72.68±1.31	70.93±2.51	0.584
HbA1c, %	8.80±2.27	8.64±2.38	0.584
Fructosamine, mmol/L	361.4±103.4	386.95±118.06	0.126
Chronic complications, n (%)	142 (66.0)	44 (49.4)	<0.01
Acute complications, n (%)	10 (4.7)	3 (3.4)	0.762
Hypoglycemia medications, n (%)			
Insulin	51 (23.7%)	11 (12.4%)	>0.05
Oral	112 (52.1%)	57 (64.0%)	
Both	52 (24.2%)	21 (23.6%)	

Data are presented as mean ± SD, number, or percentage

*P* values were calculated using *Student’s t* t test or χ2 test

**Figure 2:**
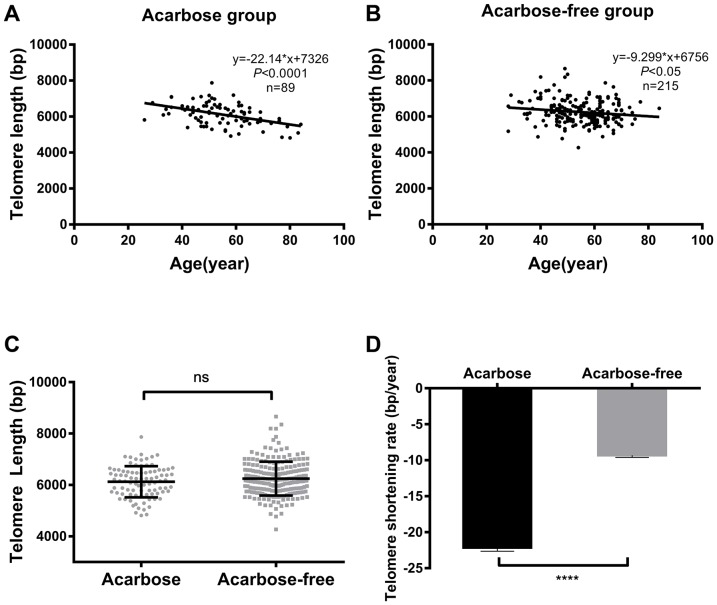
**Telomere length and TSR correlate with acarbose use in T2DM patients.** Linear regression analysis of telomere length and age in the acarbose group (**A**) and the acarbose-free group (**B**); comparison of telomere length (**C**) and TSR (**D**) between the two groups. The solid lines in **A** and **B** indicate mean telomere length, calculated by regression analysis; y = -22.14*x+7362 in **A** (*r*=-0.45, *P* <0.0001) and y = -9.29*x+6756 in **B** (*r*=-0.15, *P* <0.05) (y = telomere length in bp and x = age in years). (**D**) TSR was -22.14 ± 4.66 bp/year for the acarbose group (n = 89) and -9.29 ± 4.30 bp/year for the acarbose-free group (n = 215). Telomere length is presented as the mean ± SD. TSRs are presented as the mean ± SEM. ns indicates *P* > 0.05, ****indicates *P* < 0.0001.

Linear regression analysis showed that telomere lengths significantly shortened with age in both treatment groups (Figure 2A and 2B,* r* = -0.45,* P* < 0.0001 and *r* = -0.14, *P* < 0.05 for the acarbose and acarbose-free groups, respectively). Patients using acarbose to reduce blood glucose levels presented shorter telomere lengths (by 158.80 bp) than those who did not use acarbose, but this difference was not significant (Figure 2C). However, patients using acarbose exhibited higher TSR than patients in the acarbose-free group (-22.14 ± 4.66 bp/year *vs* -9.29 ± 4.30 bp/year respectively, *P* < 0.0001; Figure 2D).

Associations between telomere length and hypoglycemic agents, age, and incidence of chronic complications were examined using multiple linear regression. Results revealed a negative correlation between telomere length and age, hypoglycemic agents, and chronic complications (Supplementary Table 2). Telomere length decreased by 12.23 bp with each one-year increase in age. Without considering any other factors, the patients with chronic complications exhibited telomeres that were 171.80 bp shorter than those without chronic complications. Age was the most important factor affecting telomere length under all treatment conditions.

### T2DM patients taking acarbose have higher TSR than untreated patients

Because acarbose use was linked to higher TSR compared with other glycemic control regimens, we further compared baseline characteristics, telomere lengths, and TSR between the nontreatment group and the acarbose group.

The baseline data of the two groups are shown in Table 3. In line with previous findings, telomere length was negatively correlated with age in the two groups (*r* = -0.45,* P* < 0.0001 and *r* = -0.27 for the acarbose group and nontreatment group, respectively; *P* < 0.05) (Figures 2D and 1B). The patients who did not use antidiabetic agents were younger and exhibited higher levels of glycated hemoglobin and fructosamine and a greater incidence of acute complications than patients in the acarbose group (all* P* < 0.05).

**Table 3:** Patient characteristics: Acarbose vs nontreatment

Characteristic	Acarbose group	Nontreatment group	*P*
Patients, n	89	84	-
Age, years	54.2±12.5	49.10±13.25	0.011
Males, n (%)	52 (58.4)	48 (57.1)	0.879
Fasting plasma glucose, mmol/L	9.88±4.48	10.48±5.82	0.460
Postprandial plasma glucose, mmol/L	17.51±7.34	18.70±5.78	0.301
HbA1c, mmol/mol	70.93±2.51	92.02±7.76	0.000
HbA1c, %	8.64±2.38	10.57±2.86	0.000
Fructosamine, mmol/L	384.27±114.89	440.65±142.74	0.027
Chronic complications, n (%)	44 (49.4)	33 (39.3)	0.221
Acute complications, n (%)	3 (3.4)	11 (13.1)	0.025

Data are presented as mean ± SD, number or percentage

*P* values were calculated using *Student’s t* test or χ2 test

**Figure 3:**
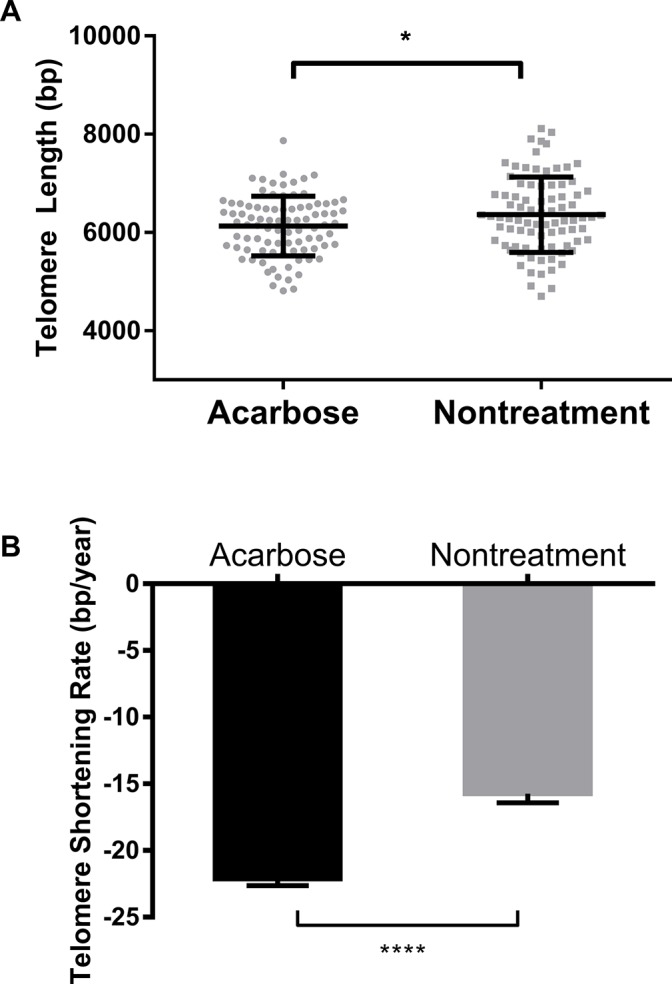
**Comparison of telomere length and TSR between acarbose-treated and nontreated T2DM patients.** Comparison of telomere length (**A**) and TSR (**B**) between the acarbose group and the nontreatment group. Telomere length is presented as the mean ± SD. TSRs are presented as the mean ± SEM. *indicates *P* < 0.05 and ****indicates *P* < 0.0001.

Telomere lengths in the acarbose group were shorter than in the nontreatment group (Figure 3A). After adjustment for age, the patients using acarbose presented also a higher TSR than patients in the nontreatment group (-22.14 ± 4.66 bp/year* vs* -15.74 ± 6.21 bp/year, respectively; *P* < 0.0001; Figure 3B).

## DISCUSSION

Telomere length is an important biomedical marker of cellular aging and is closely involved in age-related diseases such as diabetes, cancer, and cardiovascular disease [8]. Consistent with previous studies, leukocyte telomere length was negatively correlated with age in our cross-sectional analysis of 388 T2DM patients. Previous studies by You *et al*. and Blackburn et al. showed that diabetes patients lose 18–30 bp of telomere length each year [20, 26, 28, 29]. In our T2DM study population, telomere lengths were shortened by 11–16 bp/year. Our patients were Han Chinese individuals, while You *et al.* mainly analyzed postmenopausal women, and Blackburn et al. examined white American men. Our finding that T2DM patients who did not use antidiabetic agents exhibited a higher TSR than those who had taken antidiabetic agents for more than 3 months implies that antidiabetic agents may have an antiaging effect in addition to reducing blood glucose levels and complication rates. This result is similar to that reported by de Zegher *et al.*, who found that insulin sensitization via flutamide or metformin had an antiaging effect by increasing telomere length in adolescent girls with hyperinsulinemic androgen excess [30]. Our findings are also consistent with those of Uziel *et al.*, who reported that TSR is reduced with adequate glycemic control in type 2 diabetes patients [27].

Surprisingly, we found that patients whose treatment regimens contained acarbose had higher TSR than patients receiving other treatments, or no treatment at all. However, patients in the acarbose group exhibited good glycemic control compared with other antidiabetic drugs and showed better glycemic control than the nontreatment group. These results indicate that acarbose expedites telomere length shortening as age increases and that this side effect is independent of glycemic control. Several factors may be involved in acarbose’s detrimental effect on telomere length. First, acarbose reduces glucose levels by inhibiting the absorption of carbohydrates in the intestinal mucosa, whereas the fundamental mechanism of other antidiabetic drugs is to increase insulin secretion or sensitivity. Insulin’s anti-inflammatory effects include reductions in NADPH oxidase expression, ROS generation, and NF-кB binding [31]. Second, unlike acarbose, other antidiabetic agents reportedly activate phosphatidylinositol 3-kinase (PI3K) [32]. Protein kinase B (PKB), which is a downstream effector of PI3K, can phosphorylate endothelial nitric oxide synthase (eNOS) to release NO, which decreases TSR via anti-inflammatory, antioxidative, and antiapoptotic effects [32, 33]. Third, acarbose delays sucrose and starch digestion, decreasing blood glucose levels but disturbing also gastrointestinal transit, colonic function, and metabolism [34]. Many studies have demonstrated that metabolic abnormalities cause shearing stress, oxidative stress or metabolic disturbances, which could accelerate telomere shortening [35]. Finally, acarbose treatment might affect the composition of human gut bacteria species [36].

The intestinal flora is involved in the vital physiological processes of immune regulation and homeostasis of the gastrointestinal tract [37]. Changes in the intestinal flora may cause substantial changes in physiological and metabolic processes, which may accelerate cellular or organismal senescence [38]. Based on these collective findings and our multiple linear regression analysis, we conclude that acarbose use is responsible for higher TSR in T2DM patients. However, further studies are needed to identify the specific mechanism(s) underlying acarbose-mediated increases in TSR.

The strengths and limitations of our study are worth considering. This is the first study to explore the effects of antidiabetic agents on TSR and aging in patients with T2DM. Importantly, we show for the first time that people and the sample size of our study is small. Besides, the cross-sectional design of the study does not permit exploration of causality between acarbose treatment and telomere shortening, therefore, longitudinal studies will be required.

Acarbose might not be an ideal drug given its aging-promoting effect via accelerated telomere shortening. This discovery highlights that the potential impact on telomere dynamics should be considered when prescribing antidiabetic agents to T2DM patients, or other drugs for chronic disease treatment.

## MATERIALS AND METHODS

### Study subjects

### *Study population*

Between August 2012 and April 2017, 681 Han Chinese individuals were recruited randomly from inpatients of Tongji Hospital in Wuhan, Hubei. Diagnostic criteria for diabetes followed the American Diabetes Association standards [39], i.e., hemoglobin A1c ≥ 6.5%, fasting glucose ≥ 7.0 mmol/L or 2-h plasma glucose ≥ 11.1 mmol/L during an oral glucose tolerance test (OGTT). For all patients, medical history, antidiabetic agent treatment history, and family history were recorded in detail. All patients underwent a clinical examination that included plasma glucose, hemoglobin (HbA1c), insulin, and fructosamine levels. Patients with the following conditions were excluded from further analysis: (1) malignant tumors; (2) gestational diabetes mellitus; (3) stress hyperglycemia; (4) steroid-induced diabetes; (5) impaired glucose tolerance; (6) maturity-onset diabetes of the young; (7) hypophysoma; (8) thyroid nodule; (9) hyperthyroidism; (10) a recent radiation exposure history. Patients with a family history of malignant tumors or chronic diseases, such as atherosclerosis, coronary heart disease, hypertension or chronic renal failure, were also excluded to reduce the impact of confounding factors on this study. To rule out genetic influence, we selected T2DM patients older than 25 years who did not have a family history of diabetes. Patients for which analyses were unsuccessful or unreliable were also removed. Ultimately, 388 patients with T2DM were included in this research.

### *Study design*

We first divided T2DM patients into two groups. Those who had used antidiabetic drugs containing insulin or oral antidiabetic agents such as sulfonylurea, metformin, or acarbose for more than 3 months were assigned to the treatment group. Patients who had not received any medical therapy were assigned to the nontreatment group. Telomere length, TSR, and several diabetes-related test results were compared between groups.

In our preliminary study we found that patients using acarbose for glycemic control exhibited the highest TSR. To further confirm the effect of acarbose on telomere shortening, the treatment group was further subdivided into two groups: acarbose-free group (patients whose antidiabetic agents did not contain acarbose) and acarbose group (patients using acarbose for more than 3 months). Detailed information on the hypoglycemic regimens of both groups is provided in Table 2. Baseline characteristics, telomere lengths, TSR, and diabetes-related test results were compared between these two groups.

The study’s design and protocol were approved by the Ethics Committee of the Tongji Hospital of Tongji Medical College, Huazhong University of Science and Technology (IRB ID:TJ-C20160206). The procedures complied with the provisions of the Declaration of Helsinki. Informed consent was obtained from all patients.

### Sample collection

A standardized blood sample collection procedure was used across all study examination sites. Peripheral venous blood was collected into heparin tubes (4 ml; BD, NJ, USA) that were placed directly on ice and then centrifuged within 15 min of collection. Leukocyte and buffy coats were collected and stored immediately at −80°C for DNA isolation.

### Molecular and analytical determinations

### *Terminal restriction fragment length*

Genomic DNA was extracted from leukocytes following standard procedures using an AxyPrep Blood Genomic DNA Miniprep kit (Axygen, Corning, Inc., NY, USA). Hinf I (R0155L, New England Bio Labs, Beverly, MA, USA) and RsaI (R0167L, New England BioLabs) were used to digest the genomic DNA at 37°C overnight. Then, 0.7% agarose gel electrophoresis was performed to separate the digested DNA. After drying the gel, a ^32^P-labeled telomeric probe was used to detect telomeres. Subsequently, the gel was exposed to a phosphor imager and scanned with a Typhoon system (Typhoon, GE Healthcare, Wisconsin, USA) separately, and the results were visualized with Image Quant software (Molecular Dynamics, Sunnyvale, CA). The weighted mean telomere length was calculated as described previously [40].

### *Telomere shortening rate*

TSR was determined via linear regression analysis of telomere length against age [10, 41], where the slope of the linear regression represents the TSR under the assumption that inherited telomere length for all individuals does not change over time [12, 42–44].

### Statistical analysis

For continuous variables, differences between two groups were analyzed with *Student’s t* *t*-test and between multiple groups with ANOVA in normally distributed data. Non-normally distributed data were analyzed using the Mann-Whitney U test for continuous variables. Proportions of categorical data were calculated by χ^2^ test.

Pearson’s correlations analysis was performed to address the association between telomere length and age. Linear regression was also conducted to analyze the relationship between telomere length and age, and to calculate TSR. Stepwise regression analyses were applied to examine associations between age, treatment state, chronic complications, and telomere lengths.

Statistical analyses were performed using GraphPad Prism software (Version 7.0 for Windows) and SPSS (version 22.0 for Windows). Data are presented as means ± SD or means ± SEM. Significance was assessed at *P* < 0.05.

## SUPPLEMENTARY MATERIAL

Supplementary Figures

Supplementary Tables
