# Supplementary material for: Association between antidiabetic agents use and leukocyte telomere shortening rates in patients with type 2 diabetes
Source: Aging (Albany NY). 2019 Jan 28;11(2):741–55. doi: 10.18632/aging.101781 (PMC6366988; doi:10.18632/aging.101781)
Supplement: Supplementary Figures [file aging-11-101781-s001.pdf]

SUPPLEMENTARY FIGURES

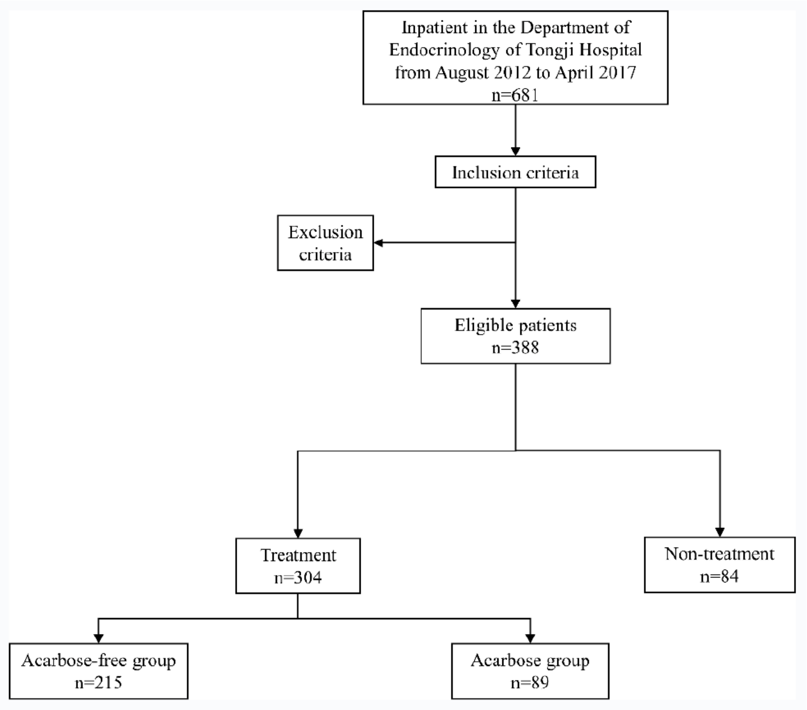

Supplementary Figure 1. The study design of this research.

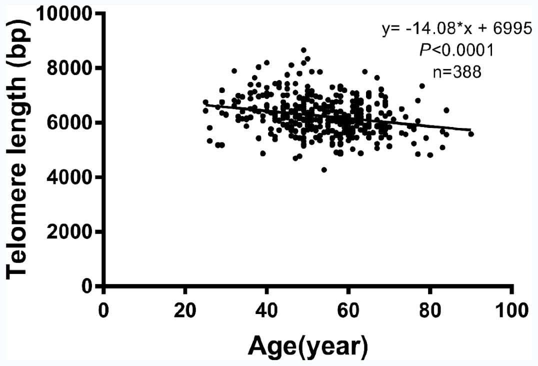

Supplementary Figure 2. Leukocyte telomere length significantly shortened with age in type 2 diabetic patients. Linear regression analysis of the relationship between the telomere length and age in type 2 diabetic patients.

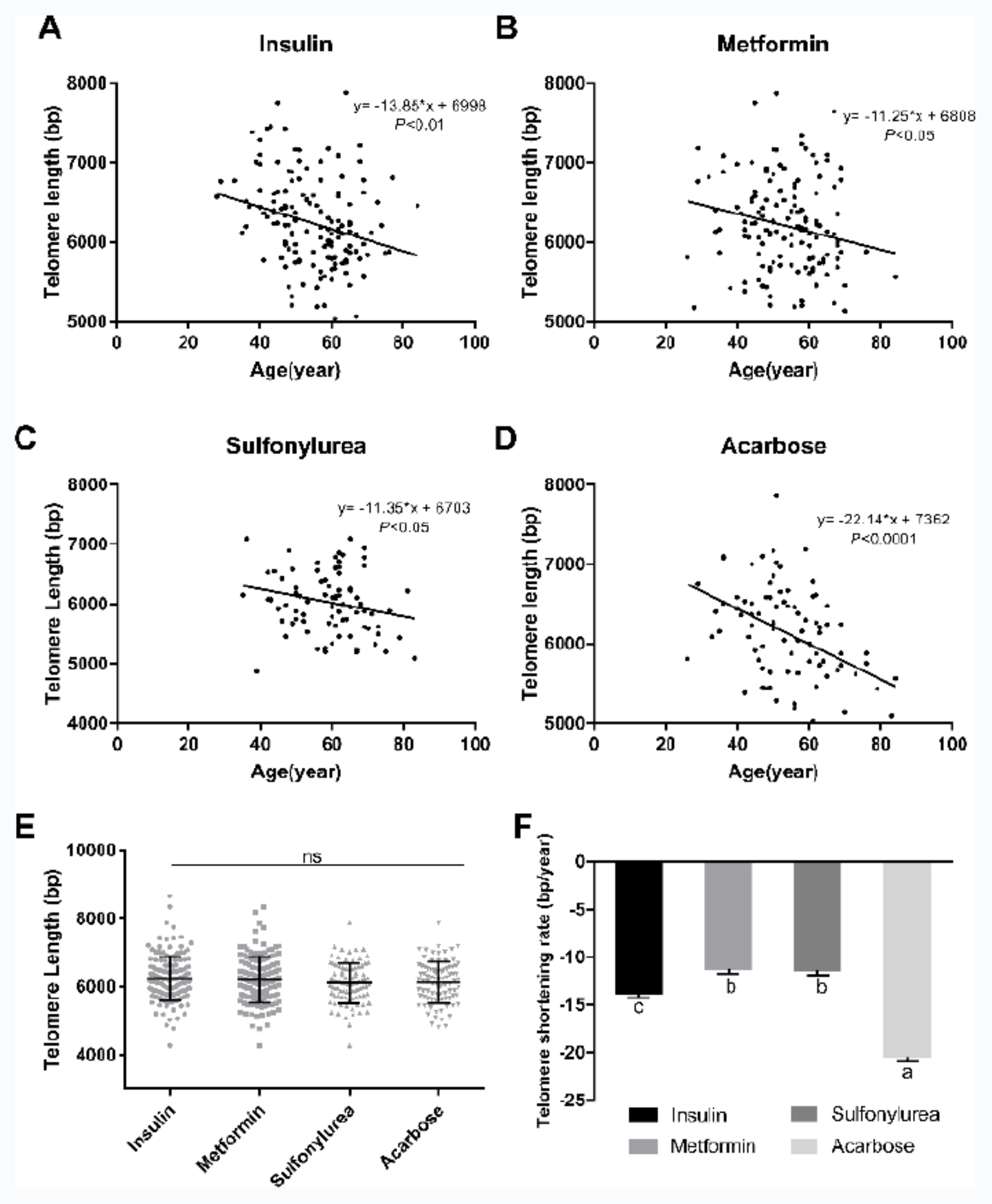

Supplementary Figure 3. Comparison of the telomere length and telomere shortening rates between various antidiabetic agents in type 2 diabetes patients. Linear regression analysis of the relationship between the telomere length and age in insulin group (**A**), metformin group (**B**), sulfonylurea group (**C**) and acarbose group (**D**). Comparison of telomere length (**E**) and telomere shortening rate (**F**) in those four groups. The telomere length was represented as the mean±SD. The telomere shortening rates were represented as the mean±SEM. In each panel, groups that share the same letter are not significantly different from each other. ns is mean *P*>0.05

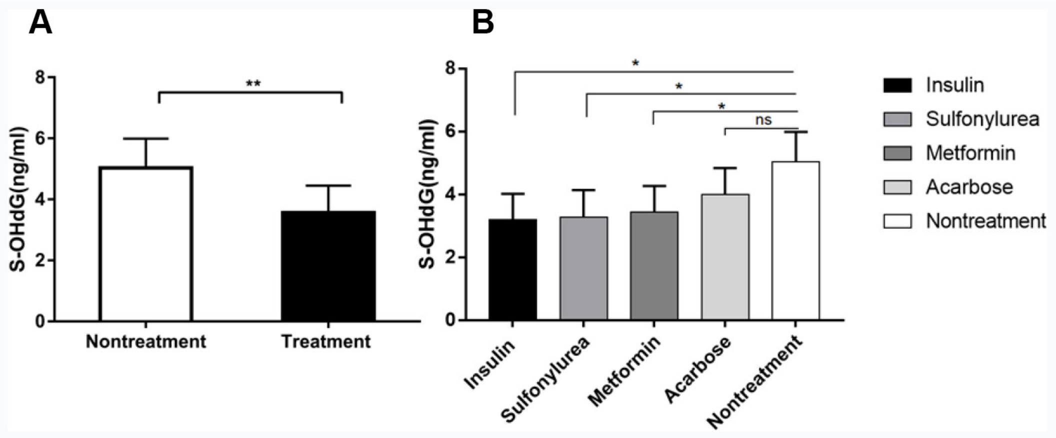

Supplementary Figure 4. The level of 8-OHdG in different groups. (**A**) the level of 8-OHdG in nontreatment and treatment groups. (**B**) the level of 8-OHdG in treatment groups with different anti-diabetic drugs and nontreatment groups. ns is *P*>0.05; * is *P*<0.05; ** is *P*<0.01

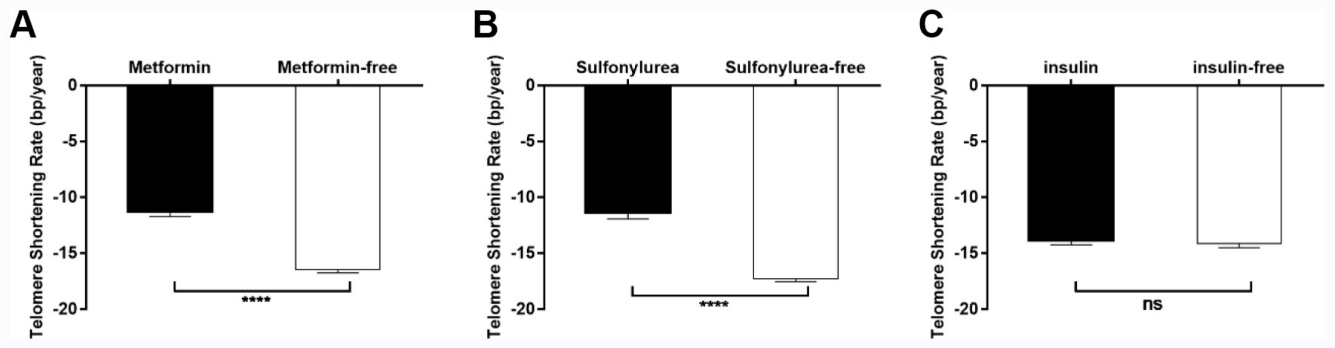

Supplementary Figure 5. The telomere shorten rate with different antidiabetic agents. (**A**) the telomere shorten rate with or without metformin. (**B**) the telomere shorten rate with or without sulfonylurea. (**C**) the telomere shorten rate with or without insulin. The telomere shortening rates were represented as the mean±SEM. **** is *P*<0.0001; ns is *P*>0.05
