# Supplementary material for: Association between antidiabetic agents use and leukocyte telomere shortening rates in patients with type 2 diabetes
Source: Aging (Albany NY). 2019 Jan 28;11(2):741–55. doi: 10.18632/aging.101781 (PMC6366988; doi:10.18632/aging.101781)
Supplement: Supplementary Tables [file aging-11-101781-s002.pdf]

SUPPLEMENTARY TABLES

Supplementary Table 1. Comparison of characteristics of participants with different antidiabetic agents.

Characteristic	Insulin group	Metformin group	Sulfonylureas group	Acarbose group	*P* value
Participants *(n)*	156	142	89	89	-
Age(year)	54.46±10.23	53.38±10.71	57.37±9.855	54.13±12.44	0.049
Male sex, n (%)	85(54.5)	80(56.3)	46(51.7)	52(58.4)	0.819
Fasting plasm glucose(mmol/L)	10.37±4.29	9.83±3.98	10.30±4.30	9.89±4.48	0.650
Postprandial plasma glucose (mmol/L)	16.94±6.30	16.08±6.06	18.54±5.67	17.52±7.34	0.081
HbA1c, (%)	9.09±2.30	8.70±2.24	8.86±2.12	8.66±2.39	0.407
Fructosamine(mmol/L)	367.42±108.26	366.07±112.32	379.78±92.98	384.27±114.89	0.657
Chronic complication, n (%)	118(75.6)	82(57.7)	65(73.0)	44(49.4)	<0.0005
Acute complication, n (%)	8(5.1)	6(4.2)	3(3.4)	3(3.4)	0.888

Data are presented as mean ± SD, number or %; *P* values were calculated using Student's t test or χ2 test

Supplementary Table 2. Multiple liner regression for the association of risk predictors with leukocyte telomere length.

		Coefficients[a]				
		Unstandardized coefficients		Standardized coefficients		
Model		β	Std. error	β	t	Sig.
1	(Constant)	6963.108	183.495		37.947	0.000
	Age	-13.805	3.291	-0.235	-4.195	0.000
2	(Constant)	6971.516	182.697		38.159	0.000
	Age	-12.297	3.366	-0.209	-3.653	0.000
	Chronic complication[b]	-148.727	76.237	-0.112	-1.951	0.052
3	(Constant)	7028.826	184.087		38.182	0.000
	Age	-12.234	3.350	-0.208	-3.652	0.000
	Chronic complication	-171.846	76.755	-0.129	-2.239	0.026
	Treatment status[c]	-158.799	80.005	-0.111	-1.985	0.048

Predictors in the Model 1: Constant), age
Predictors in the Model 2: (Constant), age, chronic complication
Predictors in the Model 3: (Constant), age, chronic complication, treatment status
[a]Dependent Variable: telomere length;
[b]diagnosed with chronic complication;
[c]using anti-diabetic agents and α-glucosidase inhibitors for glycemic control
